# Comprehensive functional evaluation of variants of fibroblast growth factor receptor genes in cancer

**DOI:** 10.1038/s41698-021-00204-0

**Published:** 2021-07-16

**Authors:** Ikuko Takeda Nakamura, Shinji Kohsaka, Masachika Ikegami, Hiroshi Ikeuchi, Toshihide Ueno, Kunhua Li, Tyler S. Beyett, Takafumi Koyama, Toshio Shimizu, Noboru Yamamoto, Fumiyuki Takahashi, Kazuhisa Takahashi, Michael J. Eck, Hiroyuki Mano

**Affiliations:** 1 Division of Cellular Signaling, National Cancer Center Research Institute, Tokyo, Japan; 2 Department of Respiratory Medicine, Graduate School of Medicine, Juntendo University, Tokyo, Japan; 3 Department of Musculoskeletal Oncology, Tokyo Metropolitan Cancer and Infectious Diseases Center Komagome Hospital, Tokyo, Japan; 4 Department of Cancer Biology, Dana-Farber Cancer Institute, Boston, MA USA; 5 Department of Biological Chemistry and Molecular Pharmacology, Harvard Medical School, Boston, MA USA; 6 Department of Experimental Therapeutics, National Cancer Center Hospital, Tokyo, Japan

**Keywords:** Cancer genomics, Oncogenes

## Abstract

Various genetic alterations of the fibroblast growth factor receptor (FGFR) family have been detected across a wide range of cancers. However, inhibition of FGFR signaling by kinase inhibitors demonstrated limited clinical effectiveness. Herein, we evaluated the transforming activity and sensitivity of 160 nonsynonymous *FGFR* mutations and ten fusion genes to seven FGFR tyrosine kinase inhibitors (TKI) using the mixed-all-nominated-in-one (MANO) method, a high-throughput functional assay. The oncogenicity of 71 mutants was newly discovered in this study. The FGFR TKIs showed anti-proliferative activities against the wild-type FGFRs and their fusions, while several hotspot mutants were relatively resistant to those TKIs. The drug sensitivities assessed with the MANO method were well concordant with those evaluated using in vitro and in vivo assays. Comprehensive analysis of published FGFR structures revealed a possible mechanism through which oncogenic *FGFR* mutations reduce sensitivity to TKIs. It was further revealed that recurrent compound mutations within FGFRs affect the transforming potential and TKI-sensitivity of corresponding kinases. In conclusion, our study suggests the importance of selecting suitable inhibitors against individual *FGFR* variants. Moreover, it reveals the necessity to develop next-generation FGFR inhibitors, which are effective against all oncogenic *FGFR* variants.

## Introduction

The fibroblast growth factor receptors (FGFR) family consists of four highly conserved transmembrane receptor tyrosine kinase genes (*FGFR1*–*4*). Each protein is comprised of an extracellular domain with three immunoglobulin (Ig)-like domains (IgI, IgII, and IgIII), followed by a transmembrane domain and two tyrosine kinase sub-domains. Activation of FGFR by fibroblast growth factors (FGFs) regulates survival and proliferative signaling pathways, in addition to metabolic homeostasis, endocrine functions, and wound repair^1,2^. Ligand binding to FGFRs activates several downstream signaling systems: the phospholipase Cγ (PLCγ), PI3K–AKT, and RAS–MAPK cascades^3,4^.

Genomic alterations of *FGFRs*, including gene amplification, activating mutations, and fusions play a crucial role in oncogenesis, tumor progression, and resistance to therapy with kinase inhibitors across a wide range of cancers^2,5,6^. Gene amplification of *FGFR1* occurs in approximately 10% and 10–25% of breast cancer and squamous-cell lung cancer cases, respectively, and is associated with a poor prognosis^7–12^. *FGFR2* gene amplification has been observed in 4–10% of gastric cancer cases and is related to a poor prognosis^13^. Although *FGFR3* gene amplification is not frequently reported, it is often observed with oncogenic mutations.^6,14,15^.

Somatic activating mutations of *FGFR2* and *FGFR3* are more common than those of *FGFR1*, while mutations of *FGFR4* are rarely observed in human cancer. *FGFR2* mutations are found in approximately 10% of endometrial carcinomas^6,16^, and the most common mutations in *FGFR3* are observed in the extracellular region (S249) of the protein structure^17,18^. Mutations in the kinase domain of *FGFR1* and *FGFR4* occur in gliomas and rhabdomyosarcomas, respectively^19,20^.

Gene fusions of the *FGFRs* have been reported in various types of cancer. The most common *FGFR3* fusions are with the transforming acidic coiled-coil containing protein 3 (TACC3) and have been discovered in glioblastoma, bladder cancer, and lung cancer^21–23^. *FGFR2* fusions with several fusion partners have been discovered in approximately 15% of intrahepatic cholangiocarcinoma cases^21,24^. Some *FGFR1* gene fusions have been observed in patients with 8p11 myeloproliferative syndrome^25^.

FGFR tyrosine kinase inhibitors (TKIs) can be classified into FGFR1/2/3 inhibitors, FGFR4 inhibitors, pan-FGFR inhibitors, and multikinase FGFR inhibitors^26^. Although activated FGFRs are promising therapeutic targets, inhibition of FGFR signaling by multikinase inhibitors or FGFR-selective inhibitors demonstrated limited clinical effectiveness^27–31^. Erdafitinib, a FGFR inhibitor approved by the US Food and Drug Administration, demonstrated an overall response rate of 32.2% and a progression-free survival of 5.5 months in patients with locally advanced or metastatic urothelial carcinoma with susceptible FGFR3 or FGFR2 genetic alterations^28^. Pemigatinib, another recent FDA-approved FGFR inhibitor, showed 35.5% of patients with *FGFR2* fusions or rearrangements achieved an objective response^32^. Previous studies suggested the importance of biological/clinical annotations for individual alterations within *FGFRs* to optimize the treatments for patients with such mutations^26^.

The transforming activity and drug sensitivity of 160 nonsynonymous *FGFR* mutations and ten fusion genes to eight FGFR TKIs was evaluated using the MANO method, a high-throughput functional assay developed in our laboratory^33^.

## Results

### *FGFR* mutations identified in human cancer

**Fig. 1:**
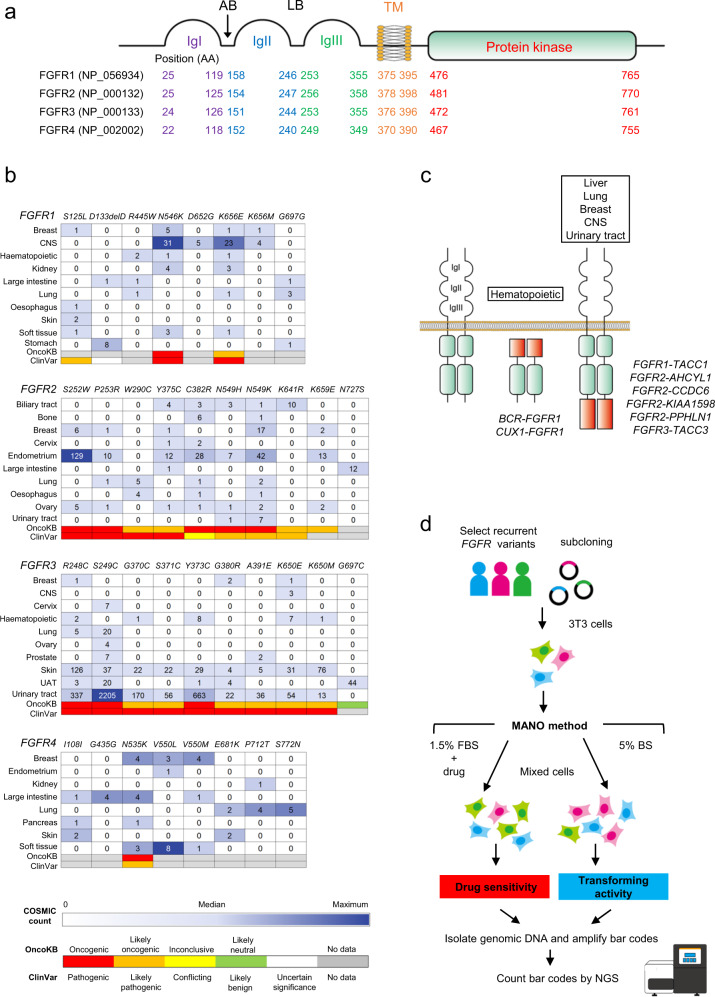
The structure of FGFR protein and the distribution of *FGFR* mutations. **a** FGFRs consist of an extracellular domain with three immunoglobulin-like domains (IgI, IgII, and IgIII), followed by a transmembrane domain and tyrosine kinase domain. An acid box (AB), which regulates FGFR interaction with partners except fibroblast growth factors (FGFs), is located between IgI and IgII. The structures of FGFR1/2/3/4 are highly conserved. **b** Mutational hotspots in *FGFRs* were observed across various types of cancer. The number of each cell indicates sample count reported in the COSMIC database. Clinical significance annotated in OncoKB and ClinVar databases were described at the bottom of the charts. The top ten major primary tissues and mutations reported in at least four samples are shown. **c** Structures of FGFR fusion proteins. FGFR fusions are classified into two types: type 1 fusion is found in hematological malignancies encoding non-transmembrane-type FGFR kinases with N-terminal substitution of fusion partner genes; type 2 fusion is common in solid tumors encoding transmembrane-type FGFRs with C-terminal substitution of fusion partner genes. Ig immunoglobulin-like domain, AB acid box, LB ligand-biding region, TM transmembrane domain, AA amino acid, CNS central nervous system, UAT upper autodigestive tract. **d** Brief overview of the MANO method. 3T3 mouse fibroblasts are infected with recombinant retrovirus expressing FGFR variants with individual 10-bp bar codes. Equal numbers of the stably transduced cells were mixed and cultured with different types of medium and/or treated with TKIs or vehicle. gDNA was harvested from the mixture of the remaining viable cells at the appropriate periods for each assay. Bar code sequences were PCR-amplified and then analyzed through deep sequencing using MiSeq sequencers to quantitate their relative abundance as a direct reflection of cell number. The read number for each bar code was normalized and compared with each other to assess transforming potential and drug sensitivity. FBS fetal bovine serum, BS bovine calf serum.

FGFRs are highly conserved transmembrane receptor tyrosine kinases, comprised of an extracellular domain with three Ig-like domains, followed by a transmembrane domain and a tyrosine kinase domain (Fig. 1a). Firstly, the prevalence of *FGFR* alterations was investigated across various cancers. In the COSMIC database (https://cancer.sanger.ac.uk/cosmic), 160 nonsynonymous mutations of *FGFR1/2/3/4* (36, 62, 41, and 25 mutations, respectively) were reported as recurrent mutations. Distinct mutational hotspots and frequent primary sites were identified in each *FGFR* (Fig. 1b). Oncogenic mutations of the *FGFR1* tyrosine kinase domain (*N546K* and *K656E*) were frequently discovered in glioma. Conversely, the *FGFR2 S252W* mutation located between IgII and IgIII, known as the ligand-biding region, was reported to be a hotspot in endometrial cancers. The most frequent mutation in *FGFRs* is the *FGFR3 S249C*, which is also located in the ligand-binding region, and known as a hotspot mutation in bladder cancer. In contrast, nonsynonymous mutations of *FGFR4* are relatively rare, although a mutation in the tyrosine kinase domain (*V550L*) was reported in rhabdomyosarcoma. The OncoKB and ClinVar data were also integrated into Fig. 1b. We further analyzed data of AACR Project GENIE^34^ using the cBioPortal. The COSMIC variant count number was well correlated with the GENIE project (Supplementary Fig. 1).

*FGFR* fusions are classified into two types (Fig. 1c); type 1 fusion is found in hematological malignancies encoding non-transmembrane-type FGFR kinases with N-terminal substitution of fusion partners, while type 2 fusion is commonly observed in solid tumors encoding transmembrane-type FGFRs with C-terminal substitution of fusion partners^35^. In this study, ten recurrent *FGFR* fusions are selected to evaluate oncogenicity and drug sensitivity.

### Transforming activity of *FGFR* variants

Transforming activity and drug sensitivity of *FGFR* variants were assessed using the MANO method. As previously reported, the MANO method is a high-throughput functional assay using Ba/F3 cells (interleukin-3 [IL-3]-dependent, murine pro-B cell line) and 3T3 cells (mouse fibroblast cell line)^33,36^. Although highly oncogenic variants of FGFRs could transform Ba/F3 cells, not all FGFR oncogenic variants did not abrogate IL-3 dependency in Ba/F3 cells. Therefore, 3T3 cells were mainly utilized to evaluate sensitivity to TKIs. PrestoBlue cell viability assay was performed using several different concentrations of fetal bovine serum (FBS) to investigate the difference in FBS dependency between the parental and FGFR variant-introduced 3T3 cells. 3T3 cells transformed by FGFR2 or FGFR4 expression maintained their proliferative capacities even at 1% FBS, whereas parental 3T3 cells showed total growth arrest (Supplementary Fig. 2). Therefore, an FBS concentration of 1.5% was chosen to evaluate the transforming activity of FGFR variants in 3T3 cells.

**Fig. 2:**
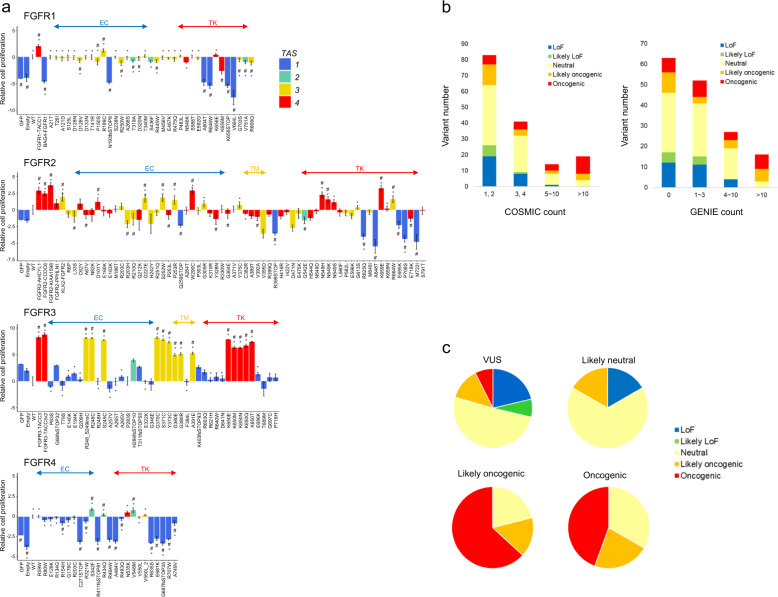
Transforming activity of *FGFR* variants. **a** Fold changes from day 3 to day 18 of 3T3 cells harboring each FGFR variant in the mixed cell population were calculated using the MANO method and shown as relative proliferation to wild-type on a base 2 logarithmic scale. Relative proliferation significantly different from those of wild-type (#) or GFP (*) are shown (paired *t*-test, *p* < 0.05). The color of each bar indicates the TAS, which are integrated assessments based on the results of the focus formation assay and low-serum cell proliferation assay. The bars are sorted according to the position of amino acids. **b** The results of the focus formation assay and growth competition assay by the MANO method were summarized to annotate the oncogenicity of variants according to the classification described in the method. The oncogenicity evaluated by the method is shown in colors and compared with the variant count number of COSMIC or AACR Project GENIE. **c** The oncogenicity evaluated by the method is shown in colors and compared with that of OncoKB. The OncoKB annotations are shown at the top of the pie chart. WT wild-type, EC extracellular domain, TM transmembrane domain, TK tyrosine kinase domain; error bars, SD.

Thus, we utilized the MANO method to compare the number of 3T3 cells expressing each FGFR variant between Day 3 and Day 18 in the assessment of the transforming potential (Fig. 2 and Supplementary Fig. 3). In parallel with the MANO method, the transforming activity of these variants was measured through the transformation activity score (TAS), which is calculated from the focus formation assay and the low-serum cell proliferation assay (Fig. 2 and Supplementary Figs. 3, 4, and 5). The results of the MANO method and TAS were highly correlated. Among the FGFR1 variants, only the oncogenic fusion FGFR1-TACC1 showed a significant growth advantage to the wild-type (WT) FGFR1. The results of FGFR2 variants indicated significant transforming activities of tyrosine kinase domain mutants (N549H and K659E), an extracellular domain mutant (W290C), and fusions (FGFR2-KIAA1598 and FGFR2-AHCYL1). Regarding FGFR4, S342F (a variant in the extracellular domain) exhibited significant oncogenicity compared with FGFR4 WT. Although the WTs of FGFR1, FGFR2, and FGFR4 showed significant transforming activity in the parental 3T3 cells, that of FGFR3 did not reveal any oncogenicity. In the transforming activity assay of FGFR3, oncogenic mutations were located in the ligand binding site (R248C and S249C), transmembrane domain (G370C, S371C, Y373C, and G380E/R), and kinase domain (K650E/M/N/Q/T) (Supplementary Fig. 5). FGFR3-TACC3 fusion also showed strong transforming activity.

The results of the focus formation assay and growth competition assay by the MANO method were summarized to annotate the oncogenicity of variants according to the following classification (Supplementary Table 1): oncogenic as higher TAS score and significantly faster growth compared with WT; likely oncogenic as higher TAS score, but not significantly faster growth compared with WT; likely loss-of-function (LoF) as lower TAS score, but not significantly slower growth compared with WT; LoF as lower TAS score and significantly slower growth compared with WT; and neutral as none of above. The oncogenicity of the variants was compared with the COSMIC count, GENIE count, and OncoKB annotation of the variant. The highly recurrent variants in the COSMIC and GENIE project were annotated as likely oncogenic or oncogenic by our functional assay, confirming the validity of the assay (Fig. 2b). Furthermore, the oncogenicity evaluated by our method was well concordant with OncoKB (Fig. 2c). Among 122 VUS, 25 variants were identified as likely oncogenic or oncogenic in this study.

The mRNA expression levels were similar among variants in previous studies using the MANO method^33,36,37^. We evaluated the mRNA and protein expression of several FGFR3 variants using real-time PCR and western blotting. While a similar level (1.0–2.5 fold change) of mRNA expression was observed, protein expressions of wild-type variant, as well as non-oncogenic variant (R248H), were low compared with those of oncogenic variants (the other variants) (Supplementary Fig. 6). This result suggested that the oncogenic mutations decrease the internalization and lysosomal degradation of FGFR3 protein.

### Sensitivity of FGFR variants to TKIs in vitro

**Fig. 3:**
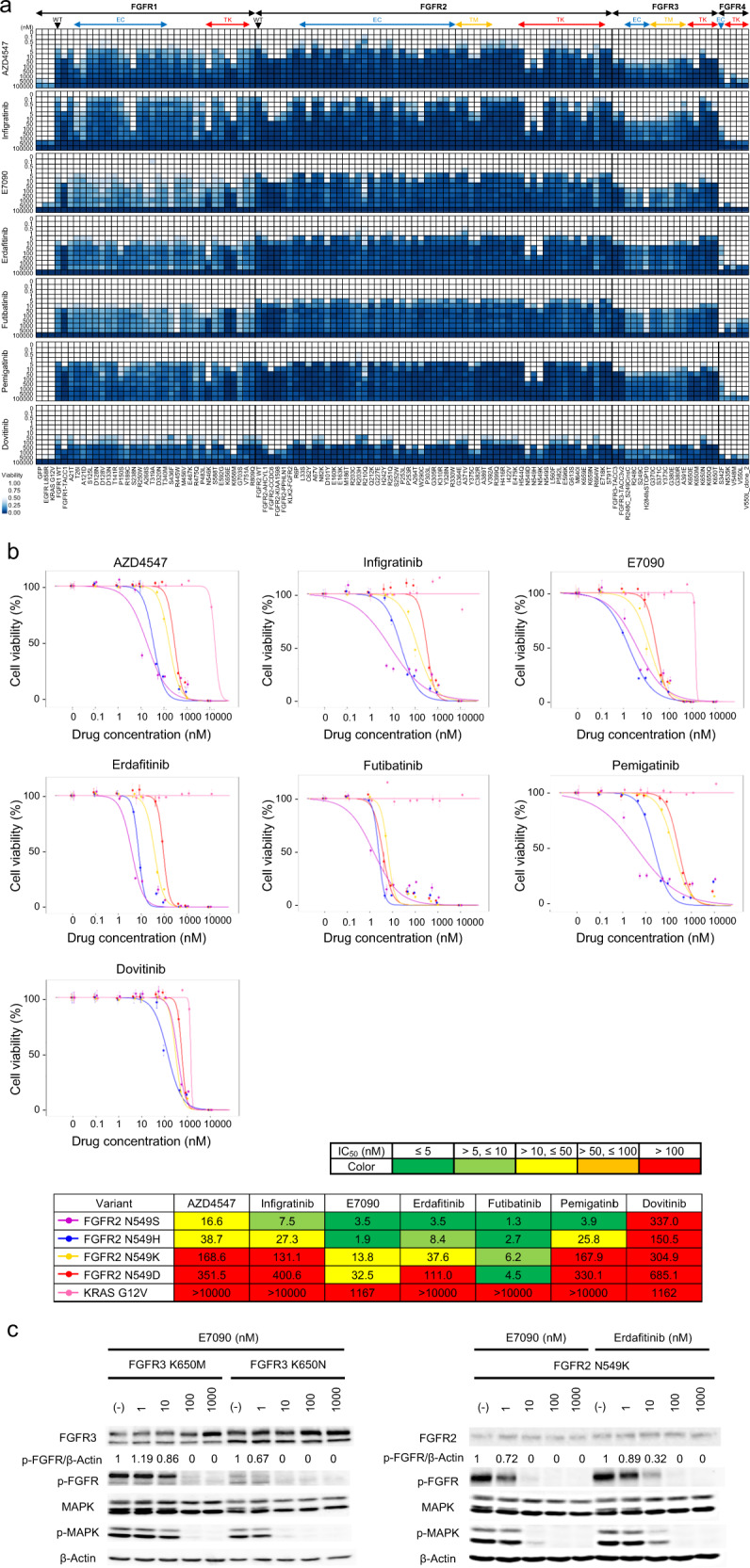
The sensitivity of FGFR variants to FGFR-targeted drugs. **a** 3T3 cells expressing FGFR variants, GFP, EGFR L858R, and KRAS G12V were treated with DMSO or FGFR-targeted drugs (AZD4547, infigratinib, E7090, erdafitinib, futibatinib, pemigatinib, and dovitinib) at the indicated concentrations. The relative viability of the treated cells with each drug versus DMSO-treated cells was measured, and the results are illustrated using the color-coded scale. **b** 3T3 cells with FGFR2 variants or KRAS G12V were incubated with the indicated concentrations of inhibitors for 5 days. Cell viability was measured using the PrestoBlue cell viability assay and plotted relative to the untreated control. Data are presented as mean ± SD (*n* = 6). The IC_50_ values of inhibitors are shown in the table at the bottom. The *p*-value in the comparison of IC_50_ between each two variants is shown in Supplementary Data 2. **c** Inhibition of phosphorylation of FGFR and downstream pathway by E7090 and erdafitinib. 3T3 cells with FGFR variants were treated with E7090 or erdafitinib for 2 h, and whole-cell lysate were analyzed by western blotting using antibodies against FGFR2, FGFR3, phospho-FGFR (p-FGFR, Tyr653/654), p44/42 MAPK, phospho-p44/42 MAPK (p-MAPK, Thr202/204), and β-Actin. WT wild-type, EC extracellular domain, TM transmembrane domain, TK tyrosine kinase domain.

**Fig. 4:**
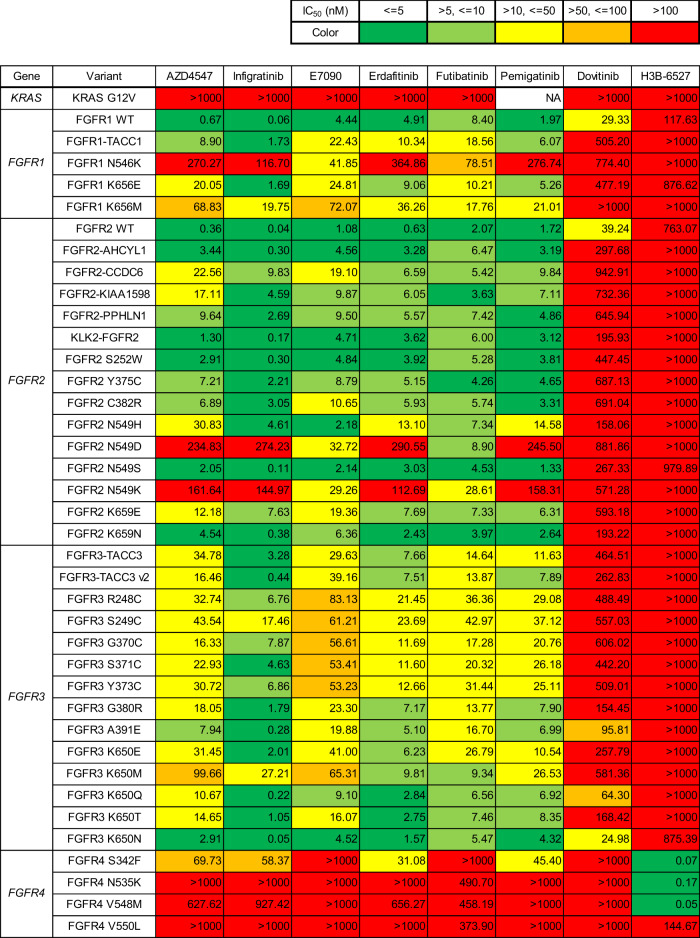
The IC_50_ values of FGFR-targeted drugs estimated using the MANO method. The IC_50_ values of FGFR-targeted drugs against FGFR variants were evaluated using the drug sensitivity assay of the MANO method in 3T3 cells.

The drug sensitivity of transformed FGFR variants was also assessed through the MANO method. The mixture of 3T3 cells expressing different types of FGFR variants were treated with eight different targeted drugs, and drug sensitivity data of 110 variants were successfully obtained (Fig. 3a). Figure 4 and Supplementary Data 1, indicate the data of common variants (COSMIC count > 10 and fusions) and that of the other variants. Most FGFR1/2 variants were sensitive to FGFR1/2/3 inhibitors (IC_50_ < 10 nM), although active mutants in the tyrosine kinase domains (FGFR1 N546K and FGFR2 N549D/K) were relatively resistant to FGFR TKIs (50 nM < IC_50_ < 500 nM). Among inhibitors, E7090 and futibatinib showed higher efficacy for those variants. Infigratinib and erdafitinib inhibited active FGFR3 mutants, including fusions, while AZD4547, E7090, pemigatinib, and futibatinib showed lower efficacy. The FGFR4 inhibitor H3B-6527 was effective against FGFR4 N535K but not against FGFR4 V550L (a known gate keeper mutation) (Supplementary Fig. 7). The multikinase inhibitor dovitinib demonstrated relatively low efficacy for all variants compared with FGFR-selective drugs.

To validate the results of the pooled assay, the respective variants to which drug sensitivity was different among TKIs were further analyzed. Interestingly, in the evaluation with the MANO method, different missense variants in the same amino acid position at FGFR2 N549 or FGFR3 K656 showed different drug sensitivities. Of note, this observation was confirmed through the PrestoBlue cell viability assay. The IC_50_ values of FGFR inhibitors at FGFR2 N549D/H/K/S was S < H < K < D, and this tendency was commonly noted among AZD4547, infigratinib and E7090 (*p* < 0.01) (Fig. 3b and Supplementary Data 2). A similar finding was observed in FGFR3 K656E/M/N/Q/T, where the IC_50_ values of AZD4547, E7090, erdafitinib, and futibatinib were N < (Q and T) < E < M (*p* < 0.01) (Supplementary Fig. 8 and Supplementary Data 2). This finding was also confirmed using Ba/F3 cells (Supplementary Fig. 9). The results showed concordance to those obtained through the MANO method (Supplementary Fig. 10).

Hierarchical clustering analysis was conducted to evaluate the similarity of FGFR inhibitors and FGFR variants using drug sensitivity data of Fig. 4 (Supplementary Fig. 11). FGFR variants were classified into four clusters. Variants in Cluster 1 were sensitive to all inhibitors, while those in Cluster 2 were relatively resistant to all inhibitors (except for E7090); variants in Cluster 2 included FGFR1 N546K and FGFR N549D/K. Cluster 3 was mainly composed of FGFR4 oncogenic variants or KRAS G12V, which were resistant to all inhibitors. Cluster 4 exhibited different sensitivity among inhibitors.

Inhibition of FGFRs and downstream signaling pathways by FGFR TKIs was evaluated through immunoblot analyses (Fig. 3c). While phosphorylation of FGFR3 K650M was suppressed by E7090 at 100 nM, that of FGFR3 K650N was decreased at lower concentrations. As shown in the right panel of Fig. 3c, FGFR2 N549K is more sensitive to E7090 than to erdafitinib. The phosphorylation status of FGFRs and MAPK and the cell viability ratios at certain concentrations of inhibitors were well correlated (Supplementary Fig. 12).

### Evaluation of the sensitivity of FGFR variants to FGFR inhibitors in vivo

**Fig. 5:**
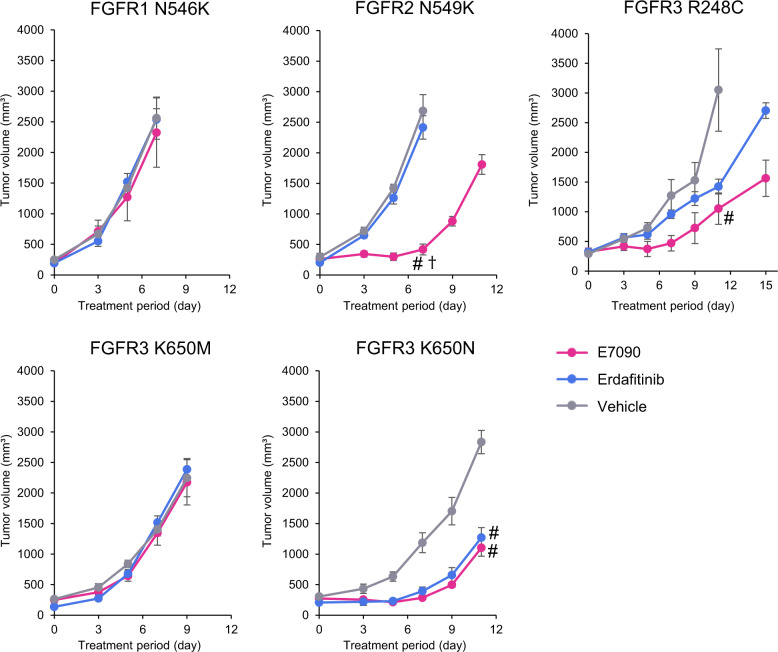
Inhibition of tumor growth in vivo by FGFR-targeted drugs. 3T3 mouse fibroblasts expressing FGFR variants were subcutaneously injected into 6-week-old female nude mice. The mice were treated with erdafitinib (12.5 mg/kg body weight), E7090 (25 mg/kg body weight), or vehicle control once daily by oral gavage (*n* = 5 mice for each group). ^#^*p* < 0.05 vs. vehicle; ^†^*p* < 0.05 vs. erdafitinib; error bars, SD.

Next, we measured the effectiveness of E7090 and erdafitinib in vivo. Mouse 3T3 fibroblasts expressing FGFR1 N546K, FGFR2 N549K, FGFR3 R248C, FGFR3 K650M, or FGFR3 K650N were injected into nude mice that were subsequently treated through oral gavage of either E7090 (25 mg/day/kg body weight), erdafitinib (12.5 mg/day/kg body weight), or vehicle control (Fig. 5). Concordant with the in-vitro data obtained from the MANO method analysis, E7090 and erdafitinib were not effective in suppressing the growth of tumors with FGFR1 N546K. In contrast, tumors with FGFR2 N549K or FGFR3 R248C exhibited a better response to treatment with E7090 versus erdafitinib. Tumor volumes of a FGFR3 K650N xenograft were significantly decreased in both drug groups compared with the vehicle group. The drug responses of FGFR2 N549K, FGFR3 K650M, and FGFR3 K650N were similar to those recorded in vitro. Interestingly, E7090 showed significant antitumor activity against tumors with FGFR3 R248C, while the IC_50_ against E7090 was approximately 83 nM according to the MANO method. None of the mice showed significant weight loss as a result of the treatment.

### Structural analysis

**Fig. 6:**
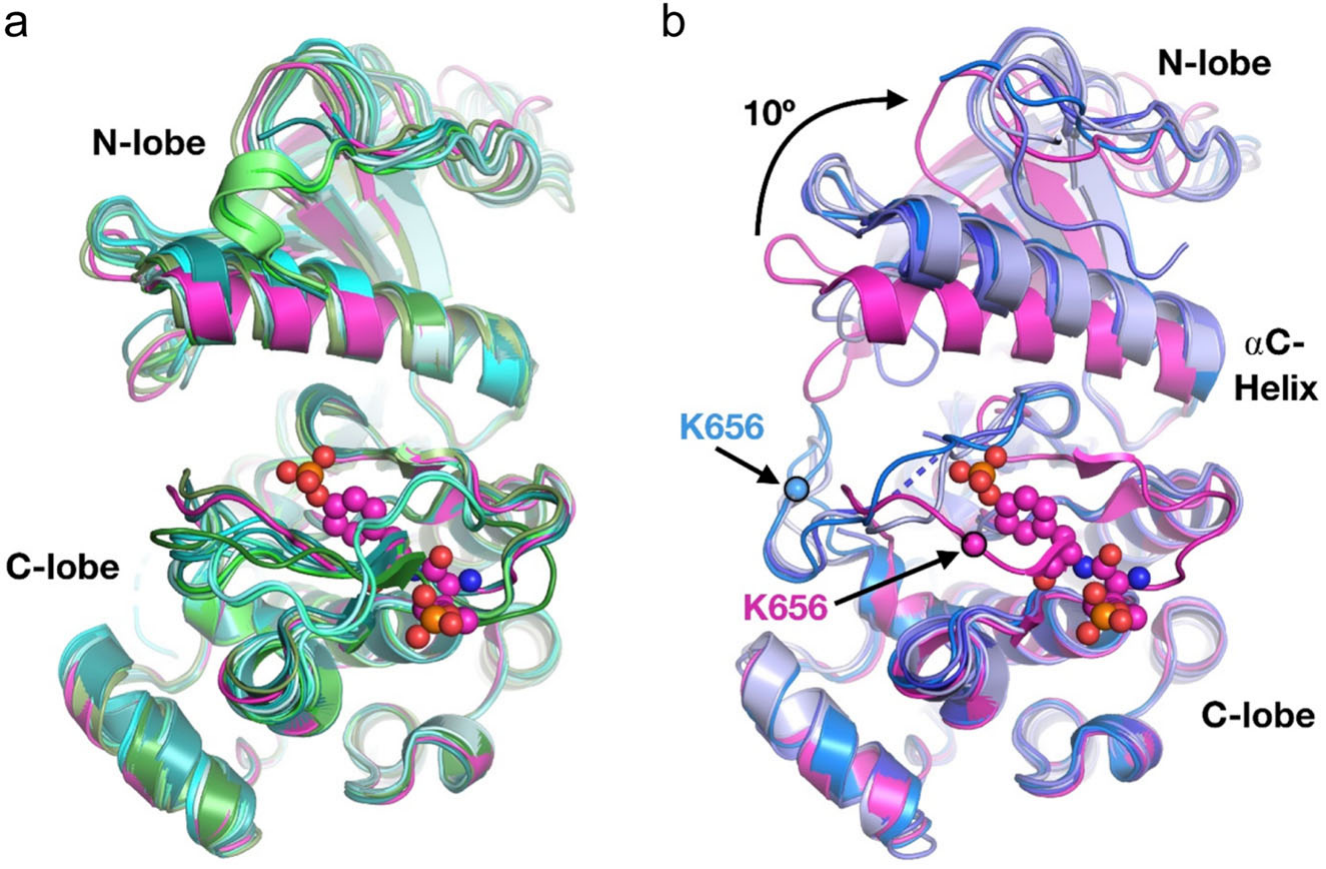
FGFR structural analysis. **a** Comparison of FGFR2 and FGFR3 kinase domain structures bearing N549H/T, K659E/N/M/Q/T, K650E, or K656E activating mutations (shades of green/teal) with wild-type FGFR1 in the active conformation (magenta). Autophosphorylated Tyr residues in the activation loop are shown in spheres. All mutant crystal structures were determined in the apo state or in the presence of an ATP analog (ball-and-stick model shown in center). Superimposed structures are from Protein Data Bank (PDB) entries 3GQI, 4K33, 2PWL, 5UHN, 2PZ5, 4J97, 5EG3, 4J98, 2PVY, 4J95, 4J99, 4J96, and 5UI0. **b** Comparison of four inhibitor-bound FGFR1 structures (shades of blue) with the structure of FGFR1 in the active conformation (magenta). In the inhibitor-bound structures, a ~10° rotation in the N-lobe was observed compared with its orientation in the active state, consistent with the inhibitor-bound structures being in the inactive, “open” conformation. The common site of mutation K656 in the activation loop also changes conformation in the inhibitor-bound structures. All structures are aligned to the C-lobe of the kinase. PDBs 3GQI, 4V05, 3TT0, 4TYI, and 5VND.

The structures of the FGFR kinase domains were aligned and analyzed to understand the mechanisms through which mutations in the kinase domain may affect sensitivity to inhibitors. Activation of FGFRs involves closure of the two lobes of the kinase domain, which is accomplished by rotation of the N-lobe and most easily visualized by examination of the αC-helix^38,39^. Kinase domains of FGFR2 and 3 with common activating mutations adopt a closed conformation similar to autophosphorylated active FGFR1 (Fig. 6a)^40^. Structures of FGFR in complex with the inhibitors AZD4547, infigratinib, dovitinib, and H3B-6527 are in a more inactive and “open” conformation not observed for FGFRs with activating mutations identified using the MANO method (Fig. 6b). Thus, inhibitor binding may require a conformation disfavored by the presence of activating mutations, leading to decreased sensitivity to inhibitors.

### Investigation of *FGFR* compound mutations

**Fig. 7:**
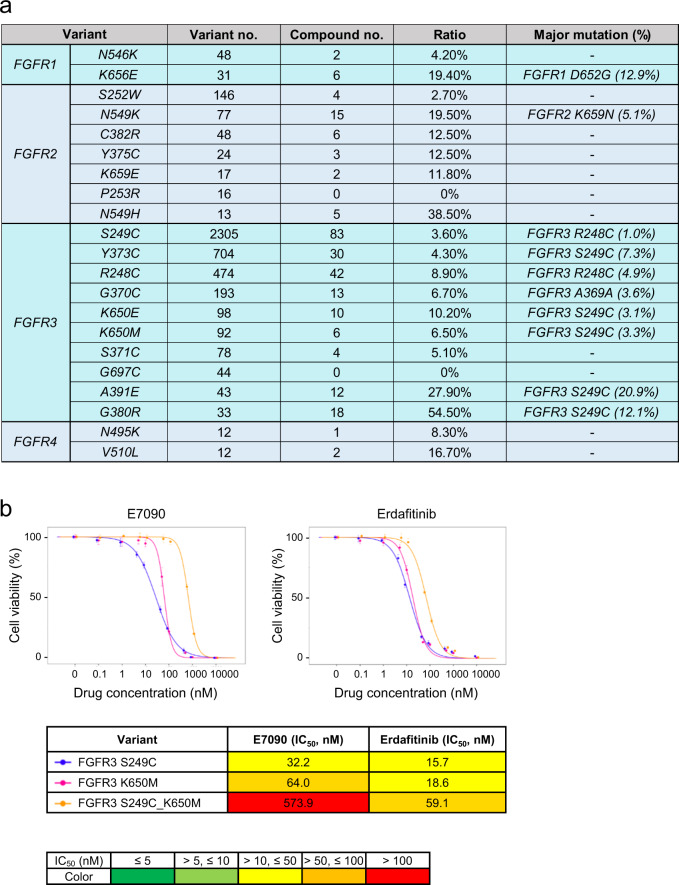
Functional analysis of *FGFR* compound mutations. **a** The frequency and patterns of *FGFR* compound mutations were investigated in the hotspot variants of *FGFRs* in the COSMIC database. **b** The drug sensitivity of *FGFR* compound mutations was evaluated with the PrestoBlue cell viability assay. 3T3 cells expressing a *FGFR* single mutation and compound mutations were treated with the indicated concentrations of E7090 or erdafitinib for 5 days. Cell viability was measured using the PrestoBlue cell viability assay and plotted relative to the untreated controls. Data are presented as the mean ± SD (*n* = 6). Estimated IC_50_ values are shown in the tables under the dose response curves. error bars, SD.

More than 400 types of *FGFR* compound mutations were observed in the COSMIC database, and 34 types of those were reported in more than two samples (Fig. 7a). The most frequent compound mutation is the combination of *S249C* and other mutations within FGFR3. The transforming activities of FGFR3 S249C compound mutations were evaluated in 3T3 cells and Ba/F3 cells (Supplementary Fig. 13a). Additional mutations on the kinase domain (FGFR3 S249C_K650E and FGFR3 S249C_K650M) showed stronger transforming activities than those of each single mutation. An additional mutation on the transmembrane domain (Y373C) with S249C did not significantly affect sensitivity to E7090 (*p* = 0.56) and erdafitinib (*p* = 0.71) (Supplementary Fig. 13b); when combined with a kinase domain mutation (S249C plus K650M or K650E), it decreased sensitivity to both agents, compared with each single mutation (Fig. 7b and Supplementary Fig. 13c, *p* < 0.05), except for S249C vs. S249C plus K650E of erdafitinib (*p* = 0.83).

Furthermore, the existence of concurrent mutations between *FGFRs* and the genes involved in different pathways, such as *PIK3CA*, *PTEN*, *AKT1/2/3*, and *MAP2K1* was investigated. Indeed, concurrent mutations with *PIK3CA* were frequently observed with *FGFR1/2/4* alterations (14.3%, 17.3%, and 38.0%, respectively) (Supplementary Data 3). Most co-mutated partners of *PIK3CA* were oncogenic mutations, such as *E545K*, *E542K*, and *H1047R*.

We also investigated whether drug efficacy is dependent on the FGFR variants in patients. For this purpose, we retrospectively collected variant information and drug efficacy data related to FGFR inhibitors in 399 cases with a FGFR gene alteration from six clinical trials investigating FGFR TKIs (Supplementary Table 2)^28,29,41–44^. Variant details were available in only 26 cases; thus, drug response was analyzed according to the types of variants, such as amplification, nonsynonymous mutations, and fusions. As a result, the overall response rate was higher in patients with mutations and fusions versus those with amplification (Supplementary Fig. 14). Four of eight patients with *FGFR3 S249C*, the most frequent mutation of *FGFR3*, exhibited a partial response or complete response to treatment with FGFR TKIs (Supplementary Data 4 and Fig. S15). The MANO method revealed that the estimated drug sensitivity of *S249C* was intermediate for all FGFR inhibitors examined. Among those clinical trials, patients with no *FGFR* alterations were eligible for NCT01703481 only. According to the clinical trial result, no responses were noted in 36 patients with unknown or no known *FGFR* alterations^43^.

## Discussion

In this study, we evaluated 170 *FGFR* variants including gene fusions; this is the most comprehensive analysis of *FGFR* mutants. Since FGFR oncogenic variants did not abrogate IL-3-dependency in Ba/F3 cells, we used mouse 3T3 cells to evaluate sensitivity to TKIs. For this purpose, we optimized the concentration of FBS in the culture medium to ensure that the growth of 3T3 cells was dependent on the activated FGFR signaling. This modified assay can expand the capability of the MANO method and conventional drug sensitivity assays to evaluate FGFR, as well as other oncogene mutants which do not transform Ba/F3 cells. We combined the focus formation assay and growth competition assay using the MANO method to annotate the oncogenicity of the variants. Among 122 VUS, 25 variants were newly identified as likely oncogenic or oncogenic in this study. Less transforming variants showing lower TAS scores and significantly slower growth than WT were evaluated as LoF variants. As the cosmic count of all LoF variants were two or three, they seem to be passenger mutations that have negative impact on tumorigenesis.

Under this condition, we demonstrated that the sensitivity of FGFRs to TKIs is dependent on each individual variant. Intriguingly, different variants at FGFR2 N549 showed different drug sensitivity but similar oncogenicity. The IC_50_ values of FGFR inhibitors at FGFR2 N549D/H/K/S were S < H < K < D, and this tendency was commonly observed among different TKIs. A similar finding was observed in FGFR3 K656E/M/N/Q/T, where the IC_50_ values of inhibitors were N < Q = T < E < M. FGFR2 N549H/K stabilizes the active conformation of the kinase by disrupting a network of hydrogen bonds, that serve as an autoinhibitory molecular break^45,46^. The K650E/M mutations of FGFR3 hamper receptor turnover and maintain the activation loop of the kinase in an active conformation^47,48^.

Among five variants performed with the in vivo sensitivity assay, the in vivo drug sensitivities of four Tyrosine kinase (TK) variants were concordant with those of in vitro assay. Interestingly, E7090 showed significant antitumor activity against tumors with FGFR3 R248C, while IC_50_ was relatively high of 83 nM against E7090 according to the in vitro MANO method. This discordance between in vitro and in vivo data may be caused by the fact that the oncogenicity of FGFR3 R248C was not as high as the other variants, and the tumor growth was slow. IC_50_ assessed by in vitro assay is evaluated in a short time (3–5 days), while in vivo drug sensitivity is usually evaluated after a longer time (up to 2 weeks, depending on the speed of tumor growth). Therefore, in vitro IC_50_ needs to be evaluated in combination with the oncogenicity of variant to apply the data into clinical practice.

Concerning the therapeutic window, the unbound average steady-state concentration of infigratinib on day 28 of Cycle 1 of the maximum tolerated dose (125 mg once daily) was 6.93 nM^42^, while that of erdafitinib was 2.5 ng/mL (equivalent to 5.6 nM)^43^. Therefore, numerous FGFR variants with predicted IC_50_ < 5 nM using the MANO method may be sensitive to these inhibitors. However, the MANO method indicated that FGFR1 N546K and FGFR2 N549D/K are resistant to AZD4547, infigratinib, erdafitinib, and pemigatinib, whereas they are relatively sensitive to E7090 and futibatinib. Indeed, Goyal et al. reported a greater clinical benefit of futibatinib against FGFR2 N549H than against N549K, which was consistent with the results of our analysis^49^. The IC_50_ values for oncogenic variants were generally higher than those recorded for the inhibition of WTs of FGFR1 and FGFR2. Therefore, the development of next-generation FGFR inhibitors which specifically inhibit all *FGFR* variants is highly desired.

The mechanisms through which mutations reduce the sensitivity to TKIs are not entirely clear, as the majority of resistant mutations do not affect the residues contacting the ATP-competitive inhibitors. However, a comprehensive analysis of published FGFR-family kinase structures indicated a possible mechanism. Activation of FGFR kinases is marked by closure of the two lobes of the kinase domain^38,39^. To promote this closure, phosphorylation of the tyrosine residues in the activation loop causes a structural shift, allowing for a ~10° rotation of the N-lobe relative to the C-lobe^40^. Reported structures of FGFR with mutations in the activation loop or “molecular break” residues are in this active “closed” conformation and display a shifted activation loop^38,39^. Additionally, these active conformation FGFR structures are all in complex with ATP analogs or in the apo state. In contrast, FGFR structures in complex with ATP-competitive inhibitors AZD4547, infigratinib, dovitinib, or H3B-6527 showed an inactive “open” conformation. Thus, a simple mechanism of resistance wherein activating mutations promote a conformation incompatible with inhibitor binding may explain the observed reduction in sensitivity to FGFR TKIs. The difference in drug sensitivity observed among variants of N549 and K650 may be attributed to the amino acids at these positions being important for kinase conformation. These amino acids may also affect access of TKIs to the adenosine triphosphate (ATP)-binding pockets.

It is noteworthy that compound mutations within *FGFRs* can change the oncogenicity and drug sensitivity to FGFR inhibitors. This is the first study evaluating the function of *FGFR* compound mutations although the recent paper mentioned the existence of *FGFR2/3* compound mutations^50^. Activation of the PI3K pathway was commonly observed and may also alter the efficacy of FGFR inhibitors. Given that recent studies reported several acquired receptor tyrosine kinase mutations after treatment with targeted drugs, evaluating the relevance of *FGFR* mutations has become increasingly important, including minor mutations, amplifications with mutations, and compound mutations.

Potential limitations of this study include the following points. Firstly, this work’s overall impact is limited by the use of 3T3 cells, where the FGFR-dependent phenotypes are unclear and not physiologically relevant. Cell type-specific and mutation-specific differences in the effects of mutant FGFR have been emphasized by previous studies^51^. However, our previous studies that evaluated EGFR and ERBB2 variants are highly correlated with the clinical data^33,36,52^. Furthermore, focus formation assay using 3T3 cells is one of the well-known methods to assess one aspect of the transforming potential of an oncogene^53^. Future clinical studies should confirm the clinical validity and utility of the assay using 3T3 to evaluate FGFR variants. Secondly, retroviral transduction of FGFR variants into cell lines results in elevated FGFR protein expression compared with endogenous FGFR. The evaluation of the transforming potential is complicated because overexpression of WT FGFR1/2/4 itself confers moderate transforming activity. Therefore, we evaluated the transforming potential of FGFR variants through TAS, which are integrated assessments based on the results of a focus formation assay and low-serum cell proliferation assay. Furthermore, three plasmids with different bar codes per one variant were constructed to obtain triplicate data in each individual assay, and may assist in compensating for the difference in copy number in gene integration. We suppose the elevated expression of FGFR3 mutants (Supplementary Fig. 6) was the result but not the cause of their oncogenic activities, because the plasmid sequence of all FGFR3 variants were the completely the same except for one or a few nucleotides at the mutations. However, it is not possible to differentiate between oncogenic activation involving an increase in specific activity and elevated activity, due to elevated expression of an FGFR mutant with no increase in specific activity over wild-type FGFR. One possible way to address this point is, for the 25 novel oncogenic variants, to validate transformation capacity in a system in which the mutants are expressed at equal or lower levels than wild-type. Thirdly, FGFRs are known to have a large number of alternate splicing forms and different isoforms of the same gene can cause different cellular responses and have different activities. Furthermore, there are 22 FGF ligands which may have hypersensitivity to cancer-specific mutations. Evaluating various splicing variants with mutations in various cellular contexts and ligand presence is needed to comprehend the precise significance of *FGFR* variants. Fourthly, the preclinical data obtained in this study are not validated by patient response; thus, these findings must be confirmed in large-scale clinical studies. Similar to the neratinib basket study for human epidermal growth factor receptor mutations^54^, the sensitivities of different types of *FGFR* mutations to FGFR-targeted TKIs should be evaluated in open basket-type clinical trials. Finally, although we identified the frequent PI3K/AKT pathway co-mutations with *FGFR* alterations and suggested the possibility of their effects on drug sensitivity, these results also need to be evaluated in the clinical setting.

In conclusion, a comprehensive evaluation of *FGFR* mutations was successfully performed using the MANO method. The structural analysis indicated that the kinase domains of FGFR2 and FGFR3 with common activating mutations adopt a closed conformation, leading to decreased sensitivity to inhibitors. This result explains why the activating mutations in the kinase domain reveal higher IC_50_ than wild types, suggesting a possible reason for the failure of clinical studies investigating FGFR TKIs to provide a drastic response in patients with *FGFR* mutant cancers. Given that each mutation of *FGFRs* exhibits different sensitivity to individual TKIs, the optimal selection of inhibitors targeted against a particular *FGFR* mutant in a patient is of critical importance. Furthermore, this study revealed that some *FGFR* mutations are resistant to any TKI. Hence, the development of next-generation FGFR inhibitors is urgently needed to overcome this resistance. The MANO method may be the strategic approach to efficiently screen such versatile TKIs. It is also desirable to develop “the rapid MANO method” that can be applied to the clinics for on-demand assessment of gene mutations found in a given tumor. The pooled phenotypic screening approach is potentially powerful, with applications to oncogenes accelerating the evaluation of the VUS of TKs and enabling the determination of the best drug for each mutation.

## Methods

### Cell lines

Human embryonic kidney (HEK) 293T cells and 3T3 mouse fibroblasts were purchased from the American Type Culture Collection (Manassas, VA, USA), and cultured in Dulbecco’s modified Eagle’s medium-F12 (DMEM-F12) supplemented with 10% FBS, 2 mmol/L glutamine, and 1% penicillin/streptomycin (all from Thermo Fisher Scientific, Waltham, MA, USA). IL-3-dependent mouse Pro-B Ba/F3 cells were cultured in a RPMI 1640 medium (Thermo Fisher Scientific) supplemented with 10% FBS, 2 mmol/L glutamine, 1% penicillin/streptomycin, and mouse IL-3 (20 U/mL; Sigma-Aldrich, St. Louis, MO, USA).

### Establishment of retroviral vector with random bar codes

The pCX6 vector was developed by inserting random 10-base-pair (bp) DNA bar code sequences upstream of the start codon of the genes of interest into the pCX4 vector^55^. The full-length WT cDNAs of human FGFR1/2/3/4 (NM_015850, NM_000141, NM_000142, and NM_002011) were cloned into the pCX6 vector. Recurrent 160 mutants of *FGFR1/2/3/4* (36, 62, 41, and 25 variants, respectively) reported in the COSMIC database v89 (https://cancer.sanger.ac.uk/cosmic) were selected for the study. FGFR variants were constructed using the QuikChange II Site-Directed Mutagenesis Kit (Agilent Technologies, Santa Clara, CA, USA) with mutation-specific primers. Ten common FGFR fusion genes were also cloned into the pCX6 vector. Fragments of fusion partner genes were constructed (Integrated DNA Technologies, Coralville, IA, USA) and joined with fragments of FGFRs using the NEBuilder HiFi DNA Assembly Master Mix (New England Biolabs, Ipswich, MA, USA). All plasmids were sequenced by Sanger sequencing to confirm the full FGFR cDNA sequence as well as 10-bp bar codes specific to each clone. Three clones with specific bar codes were constructed for each variant to obtain triplicate data in each individual assay.

### Preparation of retrovirus and gene transduction into cell lines

The recombinant plasmids were transduced together with packaging plasmids (Takara Bio, Shiga, Japan) into HEK293T cells to achieve recombinant retroviral particles. The 3T3 cells were infected in 96-well plates with ecotropic recombinant retroviruses using 4 μg/mL Polybrene (Sigma–Aldrich) for 24 h. Ba/F3 cells were seeded in retronectin-coated (Takara Bio) 96-well plates and infected with retroviruses in RPMI 1640 medium containing 20 U/mL IL-3.

### The MANO method

A schematic representation of the MANO method is shown in Supplementary Fig. 3. This method uses a retroviral vector that enables the stable integration of individual genes into the genome of assay cells (e.g., 3T3 mouse fibroblasts) along with 10-bp bar code sequences. Individually-transduced assay cells are subsequently pooled and cultured in a competitive manner to evaluate their transforming potential or drug sensitivity. At the end of the expansion period, genomic DNA were obtained from cell lysates using the QIAamp DNA Mini Kit (Qiagen, Hilden, Germany), followed by amplification by polymerase chain reaction (PCR) using primers, including indices and adapter sequences of Illumina (primer sequence is described in Supplementary Table 3). The obtained products were purified using AMPure beads (Beckman Coulter, Brea, CA, USA), and the sequencing libraries were prepared using the NEBNext Q5 Hot Start HiFi PCR Master Mix (New England Biolabs) according to the instructions provided by the manufacturer. The quality of the library was evaluated using a Qubit 2.0 fluorometer (Thermo Fisher Scientific) and the Agilent 2200 TapeStation system (Agilent). The library was sequenced on an Illumina MiSeq using the Reagent Kit V2 (300 cycles), and 150-bp paired-end reads were created (the sequencing primer loaded into the MiSeq cartridge is described in Supplementary Table 4). The bar code sequence 5′-CTAGACTGCCXXXXXXXXXXGGATCACTCT-3′ (where X denotes any nucleotide) was included in the sequencing results, and the number of each bar code in each mutant was quantified.

### Evaluation of the transforming potential using the MANO method

3T3 cells expressing various FGFR variants were mixed 3 days after mutant infection of the cells (a schema is shown in Supplementary Fig. 3b). The mixed cells were cultured in DMEM-F12 with 5% bovine serum (BS) for 18 days. Cells were passaged every 3 days, as a portion of cells were collected to count the bar codes. The assays were performed in triplicate. The cells were mixed (day 0), and cell mixtures obtained on day 3 were used as reference control for scaling the bar code count of each clone. The relative cell proliferation on a day was calculated as the ratio of the average read count across replicates of that particular day to that obtained on day 3. The relative cell proliferation of FGFR mutant cells on day 18 was compared to those of WT FGFRs and GFP expressed cells (as negative controls) using a paired *t*-test. FGFR variants with significantly high (*p* < 0.05) relative cell proliferation were regarded as activating mutants.

### Focus formation assay and low-serum cell proliferation assay

For the focus formation assay, 3T3 cells expressing various FGFR variants were cultured in DMEM-F12 supplemented with 5% bovine calf serum (BS) for 2 weeks. The cells were subsequently stained with Giemsa solution. The focus formation assay was scored as indicated: 1, no focus was observed; 2, transformed cells were partially observed; 3, diffusely transformed cells piled up in bundles; and 4, round-shaped and anchorage-independent focuses were diffusely observed. For the low-serum cell proliferation assay, cells were cultured in DMEM-F12 supplemented with 1.5% FBS for 2 weeks. Subsequently, cell proliferation was scored as indicated: 1, no viable cell was observed; 2, viable cells were partially observed; 3, transformed cells were diffusely observed; and 4, round-shaped and anchorage-independent colonies were observed. Representative images are shown in Fig. 1c.

### TAS calculation

TAS was defined by integrating all 2526 results of four experimental batches of the focus formation assay and four experimental batches of the low-serum cell proliferation assay. The ordered logistic regression model with random effects was utilized to calculate the TAS with batch-to-batch adjustment. Through this model, we obtained a tentative score (ϒ_experiment_), batch-specific (β_batch_ and *τ*_batch_), and variant-specific (*f*_variant_) random effects, and constant error variance (*ε*). We assumed that

1 $${\mathrm{{\Upsilon}}}_{{\rm{experiment}}} = {\upbeta}_{{\rm{batch}}} + \tau _{{\rm{batch}}} \times f_{{\rm{variant}}} + \varepsilon \cdot,$$

2 $${\rm{Score}}_{{\rm{experiment}}} = \left\{ {\begin{array}{*{20}{c}} {1({\mathrm{{\Upsilon}}}_{{\rm{experiment}}} \,<\, T_{1-2})} \\ {2(T_{1-2} \,<\, {\mathrm{{\Upsilon}}}_{{\rm{experiment}}} \,<\, T_{2-3})} \\ {3\left( {T_{2-3} \,<\, {\mathrm{{\Upsilon}}}_{{\rm{experiment}}} \,<\, T_{3-4}} \right)} \\ {4\left( {T_{3-4} \,<\, {\mathrm{{\Upsilon}}}_{{\rm{experiment}}}} \right)} \end{array}} \right.,$$

where Score_experiment_ was the result of an experiment; *T*_1–2_, *T*_2–3_, and *T*_3–4_ were the thresholds of the ϒ_experiment_ between four classes. Non-informative prior distributions were used for the parameters. Bayesian inference for the model was performed with the rstan package (version 2.19.2) run on R language (version 3.6.1)^56,57^. We ran four parallel chains of samplers, including 1500 warmup iterations followed by 2000 sampling iterations; all sampling iterations were adopted. Subsequently, we randomly drew 500 parameter sets, including β, *τ*, and *f* from the trace and generated 500 ϒ_variant_ values per variant with the equation ①, accounting for the batch-to-batch ratio. Finally, the TAS of a variant was defined by substituting the averaged ϒ_variant_ value into the right side of the equation ②. All the data and source codes are available in https://github.com/ikegami-tky/TAS.

### Evaluation of sensitivity to inhibitors using the MANO method

3T3 cells expressing each FGFR variant were cultured in DMEM-F12 medium with 1.5% FBS for 2 weeks. The remaining 3T3 cells were mixed and treated with the indicated concentrations (0.1 nM–10 µM) of inhibitors for 5 days. The inhibitors were one multikinase FGFR inhibitor (dovitinib), five FGFR1/2/3 inhibitors (AZD4547, infigratinib, E7090, futibatinib, and pemigatinib), one pan-FGFR inhibitor (erdafitinib), and one FGFR4 inhibitor (H3B-6527). The experiment was conducted in triplicate. We calculated the number of each bar code using the MANO method. Considering the different doubling times of the transduced cells, dimethyl sulfoxide (DMSO)-treated cell mixtures were used as the reference control for scaling the bar code count of each clone. The relative growth inhibition of each cell clone was calculated as the ratio of the average read number across triplicates to that of the DMSO control. All inhibitors used in the assay, except E7090 (provided from Eisai Co., Ltd., Tokyo, Japan), were commercially purchased: dovitinib, AZD4547, infigratinib, pemigatinib (all from MedChem Express, Monmouth Junction, NJ, USA), futibatinib (Cayman Chemical, Ann Arbor, MI, USA), and erdafitinib and H3B-6527 (both from Selleckchem, Houston, TX, USA).

### Clustering analysis

Agglomerative hierarchical cluster analysis for IC_50_ values of 36 FGFR variants to six FGFR inhibitors was performed. Every IC_50_ value was once converted into *Z*-score and logarithmically transformed. Statistical analysis was performed by R language v3.6.1 using group average linkage with Euclidean distance of log-transformed *Z*-score as a measure of similarity. The dendrogram and heatmap were generated with gplots package v3.0.3 (https://CRAN.R-project.org/package=gplots).

### PrestoBlue cell viability assay

The transformed 3T3 cells expressing each FGFR mutant were cultivated in 96-well plates in DMEM-F12 medium (100 µL of culture medium per well) with 1.5% FBS and FGFR inhibitor at concentrations ranging from 0.1 nM to 10 µM for 5 days. The transformed Ba/F3 cells expressing each FGFR mutant were cultivated in 96-well plates in RPMI 1640 medium with 20% FBS and FGFR inhibitor at concentrations ranging from 0.1 nM to 10 µM for 5 days. Subsequently, 10 μL of PrestoBlue (Thermo Fisher Scientific) was added to the plates, and the fluorescence was measured after 3 h of incubation (excitation 530 nm, emission 590 nm) at 0.1 s. The fluorescence intensities of wells without cells were used as negative controls, and dose-response curves were fit the observed cell viabilities using the “drc package” in R language^58^. The three-parameter sigmoidal function LL2.3 was used with the following settings: y0 (response without drug) = 0, robust = “mean”, method = “Nelder-Mead”. The IC_50_ was defined as the inflection point on a dose-response curve.

### Quantitative real-time PCR

The total RNA was extracted from 3T3 cells with RNA-bee reagent (Tel-Test Inc, TX, USA). RNA purification and DNase-I treatment with RNeasy Mini Kit (Qiagen), according to the instructions provided by the manufacturer. The amount of the total RNA was evaluated using NanoDrop One (Thermo Fisher Scientific). cDNA was prepared using reverse transcription reaction with SuperScript IV VILO reverse transcriptase (Thermo Fisher Scientific) using 1 µg of the total RNA from each sample, as per manufacturer’s instructions. cDNA was immediately used for quantitative real-time PCR. Real-time PCR was conducted using Power SYBR Green PCR Master Mix (Thermo Fisher Scientific) on the 7500HT Fast Real-Time PCR System (Thermo Fisher Scientific) under the following conditions: 50 °C for 2 min, 95 °C for 10 min, and 40 cycles of 95 °C for 15 s and 60 °C for 60 s. The primers specific for human FGFR3 and mouse Actb used for the real-time PCR were as follows: FGFR3: forward 5′-ACCACTGGTGCGCATCGCAAG-3′; reverse 5′-AGGGTCAGCCGGGCCCGAGAC-3′, Actb (endogenous control): forward 5′-CATTGCTGACAGGATGCAGAAGG-3′; reverse 5′-TGCTGGAAGGTGGACAGTGAGG-3′. The total RNA without reverse transcription reaction and template-free control was run in parallel to ensure the specificity of the reaction. The threshold cycle was automatically detected. The expression levels of mRNA were estimated from the linear regression equation of the calibration curve. The relative FGFR3 expression levels were normalized to that of the housekeeping gene Actb. Data are presented as relative gene expression to the sample harboring FGFR3 wild type. All reactions were run in technical triplicates.

### Western blotting

Cells were treated with indicated concentrations of inhibitors in DMEM-F12 containing 10% FBS for 2 h. Subsequently, cells were lysed in 1% NP-40 lysis buffer containing protease and phosphatase inhibitors for 15 min on ice. Cells lysates were subjected to 7.5% sodium dodecyl sulfate-polyacrylamide gel electrophoresis and immunoblotting using primary antibodies against FGFR3 (1:1000; clone C51F2), phospho-FGFR (Tyr653/654) (1:1000), p44/42 MAPK (1:1000; clone137F5), phospho-p44/42 MAPK (Thr202/204) (1:1000), and β-Actin (1:1000; clone13E5). All primary antibodies were obtained from Cell Signaling Technology (Danvers, MA, USA). The secondary antibody was horseradish peroxidase-linked anti-rabbit IgG (1:10,000, NA934V; GE Healthcare, Chicago, IL, USA). All blots were derived from the same experiment and processed in parallel.

### Xenograft tumor assays

All animal studies were conducted in accordance with the protocols approved by the Animal Ethics Committee of the National Cancer Research Center (Tokyo, Japan). Prior to injection, 3T3 cells expressing FGFR variants (1.0 × 10^6^) were mixed in phosphate-buffered saline with Matrigel (BD Biosciences, Franklin Lakes, NJ, USA) at a 1:1 ratio. Subcutaneous injection of the cell suspension was performed (200 µL per mouse) into 6-week-old female BALB/c nude mice (CLEA Japan, Tokyo, Japan). The mice were treated with erdafitinib (12.5 mg/kg body weight), E7090 (25 mg/kg body weight), or vehicle control once daily by oral gavage (*n* = 5 mice for each group). Treatment was initiated once the tumors reached a size of approximately 100–150 mm^3^. Erdafitinib was dissolved in 10% DMSO, 10% 2-hydroxypropyl-beta-cyclodextrin, and sterile ultra-pure water. E7090 was dissolved in sterile ultra-pure water. The average tumor volume in each group was calculated using the formula: *π*/6 × (large diameter) × (small diameter)^2^. The mice were sacrificed after 15 days of treatment or when the tumors reached a size of 2000 mm^3^.

### Structural analysis

FGFR structure coordinates were downloaded from the Protein Data Bank and aligned to their C-lobe (approximately residues 658–762) in PyMol (Schrödinger). The degree of rotation for the αC-helix was calculated using the C-terminal end of the helix as the fulcrum.

### Reporting summary

Further information on research design is available in the Nature Research Reporting Summary linked to this article.

## Supplementary information

Supplementary Information

REPORTING SUMMARY

Supplementary Data 1

Supplementary Data 2

Supplementary Data 3

Supplementary Data 4

## Data Availability

The authors declare that all data supporting the findings of this study are available within the paper. The sequencing data obtained in the experiments using the MANO method are not deposited in public data base because they are plasmid sequencing data; however, these data are available from the authors upon reasonable requests.

## Code availability

The references of all source codes are included within “Materials and methods”.
