# Supplementary material for: Comprehensive functional evaluation of variants of fibroblast growth factor receptor genes in cancer
Source: NPJ Precis Oncol. 2021 Jul 16;5:66. doi: 10.1038/s41698-021-00204-0 (PMC8285406; doi:10.1038/s41698-021-00204-0)
Supplement: Supplementary file 1 — Supplementary Information [file 41698_2021_204_MOESM1_ESM.docx]

**Supplementary Information**

**Comprehensive functional evaluation of variants of fibroblast growth factor receptor genes in cancer**

**Contents:**

**Supplementary Figure 1-15**

**Supplementary Table 1-4**

**Uncropped images of western blotting**

**
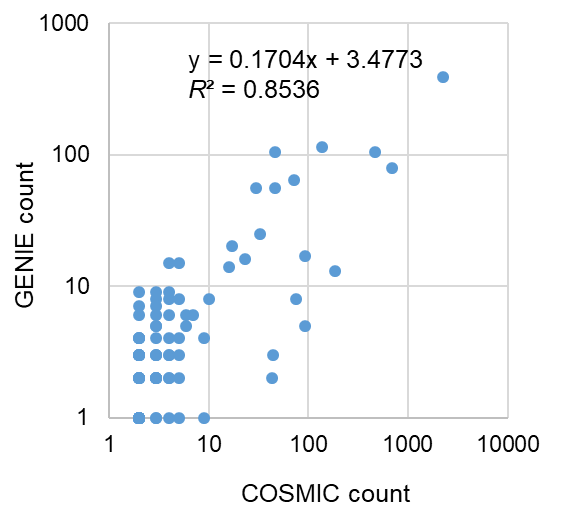
**

**Supplementary Figure 1. Variant count in GENIE and COSMIC**

The count numbers of individual *FGFR* variants in AACR Project GENIE and COSMIC are plotted.

**Supplementary Figure 2. Cell viability of 3T3 cells in medium with reduced FBS**

3T3 mouse fibroblasts expressing wild-type FGFRs or parent 3T3 cells were cultured in DMEM-F12 containing the indicated concentrations of FBS for 4 days. The PrestoBlue cell viability assay was performed to evaluate the viability of cells. *, *p* < 0.01 vs parent

**Supplementary Figure 3. Schematic representation of the MANO method**

**a,** 3T3 mouse fibroblasts are infected with recombinant retrovirus expressing FGFR variants with individual 10-bp bar codes. Equal numbers of the stably transduced cells were mixed and cultured with different types of medium and/or treated with TKIs or vehicle. gDNA was harvested from the mixture of the remaining viable cells at the appropriate periods for each assay. The bar code sequences were PCR-amplified and subjected to deep sequencing using MiSeq sequencers to quantitate their relative abundance (a direct reflection of the cell number). The read number for each bar code was normalized to that of day 3 to evaluate the transforming potential of each oncoprotein. The read number for each bar code was normalized to that of the vehicle-treated control to evaluate the inhibition profile of test compounds across transduced clones in the mixture. The transforming activity of FGFR variants in 3T3 cells was also evaluated through the focus formation assay and low-serum cell proliferation assay. **b,** Details of the cell competition assay for the evaluation of the transforming potential using the MANO method. (i) The individual FGFR variants were introduced into the 3T3 cells in 96-well plates. (ii) Subsequently, cells were harvested and passaged to large cell culture flasks. Cells with the same gene variants were mixed into the same flask. (iii) Cells were passaged every 3 days, as a portion of cells were collected for counting the bar codes. The assays were performed in triplicate. **c,** Representative images of scores 1 – 4 of the focus formation assay (upper panels) and low-serum cell proliferation assay (lower panels). The focus formation assay was scored as indicated: 1, no focus was observed; 2, transformed cells were partially observed; 3, diffusely transformed cells piled up in bundles; and 4, round-shaped and anchorage-independent focuses were diffusely observed. The low-serum cell proliferation assay was scored as indicated: 1, no viable cell was observed; 2, viable cells were partially observed; 3, transformed cells were diffusely observed; and 4, round-shaped and anchorage-independent colonies were observed.

**Supplementary Figure 4. Representative images of the focus formation assay using 3T3 cells**

The image shows 3T3 cells stained with Giemsa stain after the focus formation assay. Each variant with three different bar codes was prepared to obtain triplicate assays. The color of the columns on the right indicate the TAS of 3T3 cells. WT, wild-type.

**Supplementary Figure 5. Chronological change of 3T3 cells with FGFR variants**

3T3 cells expressing various FGFR variants were mixed and cultured in DMEM-F12 with 5% BS for 18 days. The relative proliferation was evaluated every 3 days as the fold change in the ratio of the bar code number of FGFR variants relative to day 3, which is also compensated by the value of GFP. The values are shown in a base 10 logarithmic scale. WT, wild-type.

**Supplementary Figure 6. mRNA and protein expression of FGFR3 variants**

The mRNA and protein expression of several FGFR3 variants were assessed in 3T3 that overexpressed the indicated variants by quantitative real-time PCR and western blotting. The relative expression of human FGFR3 overexpressed in 3T3 to β-Actin and endogenously expressed in 3T3 cells were evaluated and compensated with the value of wild-type FGFR3 overexpressed in 3T3.

**Supplementary Figure 7.** **Sensitivity of FGFR variants to FGFR4-targeted drugs**

3T3 cells expressing FGFR variants (GFP, EGFR L858R, and KRAS G12V) were treated with DMSO or FGFR4-targeted drug (H3B-6527) at the indicated concentrations. The relative viability of cells treated with each drug versus DMSO-treated cells was measured, and the results are illustrated using the color-coded scale. WT, wild-type; EC, extracellular domain; TM, transmembrane domain; TK, tyrosine kinase domain.

**Supplementary Figure 8.** **Sensitivity of 3T3 cells expressing FGFR3 K650 variants to FGFR-targeted drugs**

3T3 cells with FGFR3 K650 variants or KRAS G12V were incubated with the indicated concentrations of inhibitors for 5 days. Cell viability was measured using the PrestoBlue cell viability assay and plotted relative to that recorded for the untreated control. Data are presented as the mean ± SD (n = 6). The IC_50_ values of inhibitors are shown in the table at the bottom. The *p*-value in the comparison of IC_50_ between each two variants is shown in Supplementary Table 3.

**Supplementary Figure 9.** **Sensitivity of Ba/F3 cells expressing FGFR3 K650 variants to FGFR-targeted drugs**

Ba/F3 cells with FGFR3 K650 variants or KRAS G12V were incubated with the indicated concentrations of inhibitors for 5 days. Cell viability was measured using the PrestoBlue cell viability assay and plotted relative to that recorded for the untreated control. Data are presented as the mean ± SD (n = 6). The IC_50_ values of inhibitors are shown in the table at the bottom. The *p*-value in the comparison of IC_50_ between each two variants is shown in Supplementary Table 3.

**Supplementary Figure 10. Concordance in sensitivity to TKIs assessed through the MANO method and PrestoBlue cell viability assay**

Comparison of cell viability measured with the PrestoBlue cell viability assay and MANO method for 3T3 cells with nine FGFR mutants following treatment with seven inhibitors shown in Fig. 3B and Supplementary Fig. 8. Each data point was normalized to data obtained from vehicle-treated cells. Pearson’s correlation coefficient (*r*) was calculated as 0.92 (*p* < 0.001). The low ratio area is magnified in the right panel.

**Supplementary Figure 11. Clustering analysis using drug sensitivity data**

Hierarchical clustering with Euclidean distance was conducted to evaluate the similarity of FGFR inhibitors and FGFR variants using drug sensitivity data. The color of each cell indicates Z-score of natural log-transformed IC_50_ values. Dendrogram was generated based on group average values.

**Supplementary Figure 12. The correlation between the phosphorylation of FGFR and cell viability**

The IC_50_ values for the phosphorylation of FGFRs using the western blotting data and for the cell viability of 3T3 cells are compared with respective FGFR variants. The coefficient of determination (*R*^2^) between both IC_50_ values was 0.72.

**Supplementary Figure 13. Transforming activity and drug sensitivity of *FGFR* compound mutations**

**a,** Oncogenic potentials of *FGFR* compound mutations shown in the left table were assessed by introducing FGFR compound variants into 3T3 cells and Ba/F3 cells. We defined the transforming activity of Ba/F3 by the period to acquire IL-3 independency: 1, do not show transformation at 3 weeks after GENIEntroduction; 2, transform between 2 to 3 weeks; 3, transformation observed between 1 to 2 weeks; and 4, transformation observed within 1 week. The 3T3 focus formation assay was scored as indicated: 1, no focus was observed; 2, transformed cells were partially observed; 3, diffusely transformed cells piled up in bundles; and 4, round-shaped and anchorage-independent focuses were diffusely observed. The image on the right shows 3T3 cells stained with the Giemsa stain after the focus formation assay. **b, c,** Drug sensitivity of FGFR compound variants evaluated with the PrestoBlue cell viability assay. 3T3 cells expressing FGFR3 S249C, Y373C, K650E, and their compound mutation FGFR3 S249C_Y373C and S249C_K650E were treated with indicated concentrations of E7090 or erdafitinib for 5 days. Cell viability was measured using the PrestoBlue cell viability assay and plotted relative to that recorded for the untreated controls. Data are presented as the mean ± SD (n = 6). Estimated IC_50_ values are shown in the table under the dose response curves. error bars, SD

**Supplementary Figure 14. Summary of the best tumor response of patients treated with FGFR-targeted drugs in five clinical trials**

The best tumor response of patients treated with FGFR-targeted drugs in five clinical trials (Supplementary Table 5 and 6) was summarized for the following variant types: FGFR amplification (**a**), FGFR mutation (**b**), and FGFR fusion (**c**). Assessment of responses of solid tumors was performed according to the Response Evaluation Criteria in Solid Tumors version 1.0 or 1.1 at the period of each protocol. CR, complete response; PR, partial response; SD, stable disease; PD, progressive disease.

**Supplementary Figure 15. Computed tomography images indicate the antitumor activity of ASP5878**

A 67-year-old female patient with metastatic bladder cancer harboring the FGFR3 mutation S249C achieved a partial response to ASP5878, a selective small-molecule inhibitor of FGFRs, against target legions of multiple lung and bladder metastases. **a,** At baseline. **b,** After four cycles. Blue and yellow arrows show right lung and bladder lesions, respectively. The patient discontinued treatment after four cycles (3 months) due to ocular toxicity (grade 2 corneal damage). The ocular toxicity was reversible after termination of treatment with a FGFR inhibitor.

**Supplementary Table 1. Summary of oncogenicity of FGFR variants**

WT, wild type; NA, not applicable; TAS, transformation activity score; LoF, loss-of-function.

**Supplementary Table 2. Summary of the clinical trials investigating FGFR-targeted drugs**

**Supplementary Table 3. Primer sequence for the bar code amplification**

**Supplementary Table 4. Primer sequence for next-generation sequencer analysis**

**Uncropped images of western blotting**
