# Supplementary material for: Comprehensive functional evaluation of variants of fibroblast growth factor receptor genes in cancer
Source: NPJ Precis Oncol. 2021 Jul 16;5:66. doi: 10.1038/s41698-021-00204-0 (PMC8285406; doi:10.1038/s41698-021-00204-0)
Supplement: Supplementary file 2 — REPORTING SUMMARY [file 41698_2021_204_MOESM2_ESM.pdf]

nature research

Corresponding author(s): Shinji Kohsaka
Hiroyuki Mano

Last updated by author(s): 2021/03/17

Reporting Summary

Nature Research wishes to improve the reproducibility of the work that we publish. This form provides structure for consistency and transparency in reporting. For further information on Nature Research policies, see our Editorial Policies and the Editorial Policy Checklist.

Statistics

For all statistical analyses, confirm that the following items are present in the figure legend, table legend, main text, or Methods section.

n/a	Confirmed	
☐	☒	The exact sample size (n) for each experimental group/condition, given as a discrete number and unit of measurement
☐	☒	A statement on whether measurements were taken from distinct samples or whether the same sample was measured repeatedly
☐	☒	The statistical test(s) used AND whether they are one- or two-sided<br>*Only common tests should be described solely by name; describe more complex techniques in the Methods section.*
☒	☐	A description of all covariates tested
☐	☒	A description of any assumptions or corrections, such as tests of normality and adjustment for multiple comparisons
☐	☒	A full description of the statistical parameters including central tendency (e.g. means) or other basic estimates (e.g. regression coefficient) AND variation (e.g. standard deviation) or associated estimates of uncertainty (e.g. confidence intervals)
☐	☒	For null hypothesis testing, the test statistic (e.g. F, t, r) with confidence intervals, effect sizes, degrees of freedom and P value noted<br>*Give P values as exact values whenever suitable.*
☐	☒	For Bayesian analysis, information on the choice of priors and Markov chain Monte Carlo settings
☐	☒	For hierarchical and complex designs, identification of the appropriate level for tests and full reporting of outcomes
☐	☒	Estimates of effect sizes (e.g. Cohen's d, Pearson's r), indicating how they were calculated

Our web collection on statistics for biologists contains articles on many of the points above.

Software and code

Policy information about availability of computer code

Data collection	No software was used.
Data analysis	The source codes of TAS score calculation is available in https://github.com/ikegami-tky/TAS.The dendrogram and heatmap are generated with gplots package v3.0.3 (https://CRAN.R-project.org/package=gplots).

For manuscripts utilizing custom algorithms or software that are central to the research but not yet described in published literature, software must be made available to editors and reviewers. We strongly encourage code deposition in a community repository (e.g. GitHub). See the Nature Research guidelines for submitting code & software for further information.

Data

Policy information about availability of data

All manuscripts must include a data availability statement. This statement should provide the following information, where applicable:

- Accession codes, unique identifiers, or web links for publicly available datasets
- A list of figures that have associated raw data
- A description of any restrictions on data availability

The authors declare that all data supporting the findings of this study are available within the paper.

Field-specific reporting

Please select the one below that is the best fit for your research. If you are not sure, read the appropriate sections before making your selection.

☒ Life sciences ☐ Behavioural & social sciences ☐ Ecological, evolutionary & environmental sciences

For a reference copy of the document with all sections, see nature.com/documents/nr-reporting-summary-flat.pdf

Life sciences study design

All studies must disclose on these points even when the disclosure is negative.

Sample size	No sample size calculation was performed.
Data exclusions	No data exclusions were performed.
Replication	For MANO method, three biologically independent experiments were performed. Seven drugs used in cell viability assay and MANO method showed similar results.<br>For real-time qPCR, three biological replicates were performed, and each with three technical replicates.<br>All the repeated experiments showed similar results. The number of replication for each experiment was described in figure legends.<br>For cell viability assay, six technical replicates for each variant were performed.<br>Reproducibility of cell viability assay was confirmed by two independent experiments.<br>Reproducibility of western blotting was confirmed by two independent experiments.
Randomization	Randomization was not performed for this study.
Blinding	Blinding in this study was not performed.<br>All in vitro experiments whose data were generated by machine reading, blinding or not won't affect the results.<br>Data analysis were performed by the computational pipeline.

Reporting for specific materials, systems and methods

We require information from authors about some types of materials, experimental systems and methods used in many studies. Here, indicate whether each material, system or method listed is relevant to your study. If you are not sure if a list item applies to your research, read the appropriate section before selecting a response.

Materials & experimental systems

n/a	Involved in the study	
☐	☒	Antibodies
☐	☒	Eukaryotic cell lines
☒	☐	Palaeontology and archaeology
☐	☒	Animals and other organisms
☒	☐	Human research participants
☒	☐	Clinical data
☒	☐	Dual use research of concern

Methods

n/a	Involved in the study	
☒	☐	ChIP-seq
☒	☐	Flow cytometry
☒	☐	MRI-based neuroimaging

Antibodies

Antibodies used	Primary antibodies against FGFR3 (1:1,000; clone C51F2), phospho-FGFR (Tyr653/654) (1:1,000), p44/42 MAPK (1:1,000; clone137F5), phospho-p44/42 MAPK (Thr202/204) (1:1,000), and β-Actin (1:1,000; clone13E5) were obtained from Cell Signaling Technology (Danvers, MA, USA). The secondary antibody was horseradish peroxidase-linked anti-rabbit IgG (1:10,000, NA934V; GE Healthcare, Chicago, IL, USA).
Validation	All antibodies used in western blotting are obtained from commercial sources. Please see manufacturer's link for validation of antibody.

Eukaryotic cell lines

Policy information about cell lines

Cell line source(s)	Human embryonic kidney (HEK) 293T cells and 3T3 mouse fibroblasts were purchased from the American Type Culture Collection (Manassas, VA, USA)
Authentication	Cells were not authenticated by the authors.

Mycoplasma contamination	Cells were not tested for mycoplasma contamination.
Commonly misidentified lines (See ICLAC register)	No commonly misidentified cell lines were used.

Animals and other organisms

Policy information about studies involving animals; ARRIVE guidelines recommended for reporting animal research

Laboratory animals	6-week-old female BALB/c nude mice were purchased from CLEA Japan (Tokyo, Japan).
Wild animals	No wild animals were used.
Field-collected samples	No samples were collected from the field.
Ethics oversight	All animal studies were conducted in accordance with the protocols approved by the Animal Ethics Committee of the National Cancer Research Center (Tokyo, Japan).

Note that full information on the approval of the study protocol must also be provided in the manuscript.
